# Insight into Green Extraction for Roselle as a Source of Natural Red Pigments: A Review

**DOI:** 10.3390/molecules28031336

**Published:** 2023-01-31

**Authors:** Dwila Nur Rizkiyah, Nicky Rahmana Putra, Mohd Azizi Che Yunus, Ibham Veza, Irianto Irianto, Ahmad Hazim Abdul Aziz, Sri Rahayuningsih, Erny Yuniarti, Ikhwani Ikhwani

**Affiliations:** 1 Centre of Lipid Engineering and Applied Research (CLEAR), Ibnu Sina Institute for Scientific and Industrial Research, UTM Johor Bahru, Universiti Teknology, Johor Bahru 81310, Malaysia; 2 Department of Mechanical Engineering, Universiti Teknologi PETRONAS, Seri Iskandar 32610, Malaysia; 3 Department General Education, Faculty of Resilience, Rabdan Academy, Abu Dhabi 22401, United Arab Emirates; 4 Faculty of Food Science and Nutrition, Universiti Malaysia Sabah, Kota Kinabalu 88400, Malaysia; 5 National Research and Innovation Agency, Jakarta 12710, Indonesia

**Keywords:** supercritical carbon dioxide, extraction, roselle, red pigments, anthocyanin

## Abstract

Roselle (*Hibiscus sabdariffa* L.) is a source of anthocyanins as red pigments that is extensively farmed in tropical and subtropical regions, including Indonesia, Malaysia, China, Thailand, Egypt, Mexico, and West India. The roselle plant contains a variety of nutrients, including anthocyanins, organic acids, pectin, etc. Due to the toxicity and combustibility of the solvents, traditional extraction methods for these compounds are restricted. Obtaining pure extracts is typically a lengthy procedure requiring many processes. Supercritical carbon dioxide (ScCO_2_) extraction as a green technology is rapidly improving and extending its application domains. The advantages of this method are zero waste production, quicker extraction times, and reduced solvent consumption. The ScCO_2_ extraction of natural pigments has great promise in food, pharmaceuticals, cosmetics, and textiles, among other uses. The ScCO_2_ technique for natural pigments may also be advantageous in a variety of other contexts. Due to their minimal environmental risk, the high-quality red pigments of roselle rich in anthocyanins extracted using ScCO_2_ extraction have a high sustainability potential. Therefore, the objective of this review is to increase knowledge related to the natural colorant of roselle as a substitute for chemically manufactured colorants using ScCO_2_ as a green method. This article covers ScCO_2_ extraction, particularly as it relates to the optimization of pigments that promote health. This article focuses on the high extraction efficiency of ScCO_2_ extraction. Natural colorants extracted via ScCO_2_ are regarded as safe compounds, especially for human consumption, such as novel functional food additives and textile and pharmaceutical colors.

## 1. Introduction

Roselle is one of the potential sources of anthocyanins as red pigments [1]. Roselle is cultivated widely in tropic and sub-tropic countries, such as Indonesia, Malaysia, China, Mexico, Thailand, and Egypt [2]. Roselle is brightly red colored and rich in nutrients such as organic acid, anthocyanins, and pectin. Grajeda-Iglesias, et al. [3] also identified that roselle is rich in anthocyanins as red natural pigments. Cyanidin 3-glucoside and delphinidin 3-glucoside are the most abundant anthocyanins present in the calyces of roselle [4].

Anthocyanins categorized as flavonoids are water-soluble pigments that can be found in most vascular plants. Anthocyanins have been revealed as one of the most promising ingredients in the beverage, cosmetics, food, and nutraceutical industries. This bioactive compound can improve the control of Type II diabetes and coronary heart disease and prevent cancer development [5]. Anthocyanins as high polar compounds are found predominantly in inner cell layers such as the epidermis and peripheral mesophyll cells [6,7,8,9,10].

**Figure 1:**
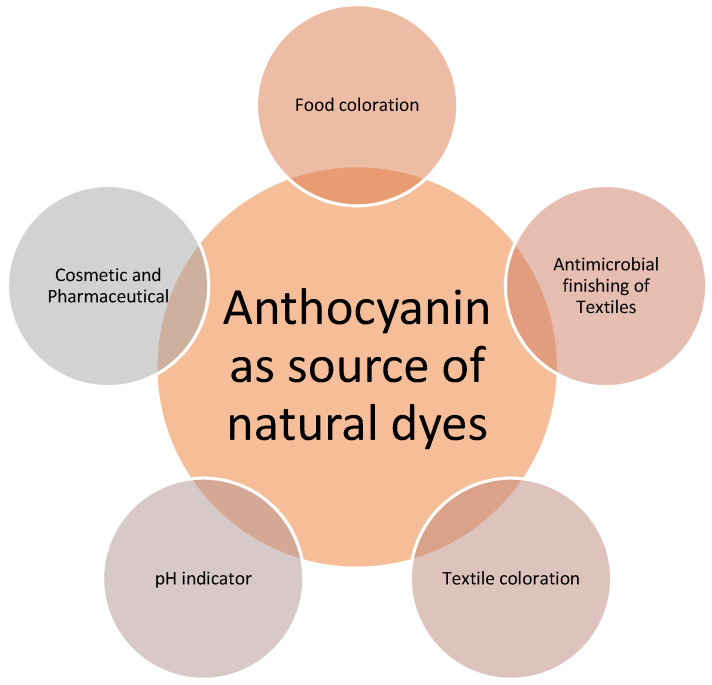
Various applications of anthocyanins as natural dyes.

In response to a growing awareness of the detrimental environmental effects of synthetic products, the use of sustainable natural products has become more important [11]. To produce great extraction yields from natural materials, it is vital to choose the most suited extraction techniques and circumstances. Natural colorants have gained popularity due to their properties and health-promoting advantages, as well as their low cytotoxicity in comparison to synthetic colorants [12]. These natural colorants have a wide range of potential uses, notably in the food, textile, and pharmaceutical industries as shown in Figure 1 [13,14,15].

Supercritical carbon dioxide (ScCO_2_) is often used to extract bioactive compounds, such as phenolic, flavonoid, and antioxidant compounds [16,17,18]. This method is a novel approach for optimizing the extraction of chemicals of interest, especially for anthocyanins since it is a non-hazardous and eco-friendly solvent. Mohd-Nasir, et al. [19] and Abdul Aziz, et al. [20] reported that this technology has many benefits over traditional extraction techniques, including a reduction in extraction time, an improvement in extract quality, reduced extraction agent costs, and an eco-friendly operation. This technique is rapidly gaining acceptance as an alternative to solid sample extraction [21,22,23]. It is also relatively recent and quite promising [24].

However, pure carbon dioxide cannot be applied to extract anthocyanins from roselle [11,25,26,27,28,29,30]. Commonly, ethanol is applied as an entrainer/modifier for supercritical carbon dioxide to extract anthocyanins. According to the United Nations’ sustainable development goals for good health and well-being, ethanol is classified as a generally recognized as safe (GRAS) solvent, as it belongs to Class 3 in the Guidance for Industry of the US Food and Drug Administration (FDA), which means it is considered non-hazardous.

This review contrasts the ScCO_2_ approach with conventional extraction techniques to highlight the benefits and drawbacks of ScCO_2_ in comparison to conventional methods. In addition, the concepts and mechanics of ScCO_2_ are described, as well as the evolution of ScCO_2_ technology up to the current day. The optimization of several extraction parameters, such as the co-solvent ratio, flow rate, extraction time, temperature, raw matrix, and pressure, are examined in depth. This article also included anthocyanins as red pigments for natural colorants. In addition, the appropriate ScCO_2_ extraction conditions are presented, along with a prospective outlook on the commercialization of roselle as a natural colorant source.

## 2. Anthocyanins as Red Pigments

Due to their great stability (light, oxygen, heat, and pH), homogeneity, coloring power, independence from microbial contamination, and comparatively cheap manufacturing cost, synthetic colorants are gradually replacing natural colorants. However, the usage of synthetics is decreasing progressively owing to their possible health risks [31]. The issue surrounding the use of chemicals in consumables is rooted in the tension between technical need and safety, as well as the advantages and possible toxicological dangers of long-term exposure to these compounds [32]. It has been observed that the growth in consumer understanding about what they eat is the primary reason in lowering the usage of synthetic chemicals [14]. In addition, studies have highlighted the toxicity of food colorings since 1960, which has aroused grave worry among global health authorities (Table 1).

**Figure 2:**
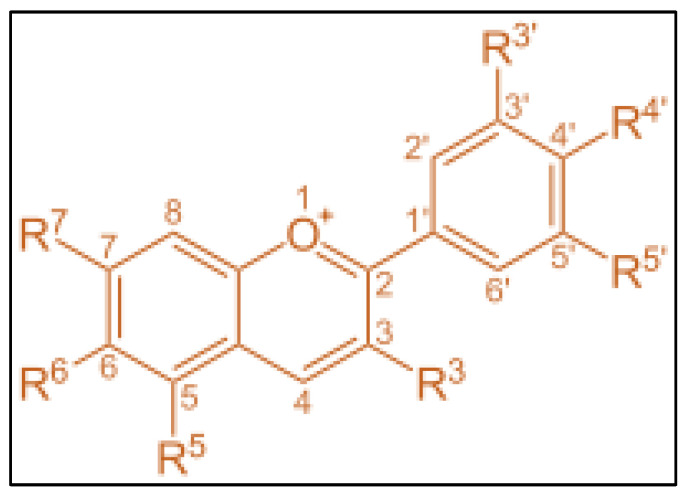
Structure of anthocyanins.

Anthocyanins can be obtained from various plant sources such as tea, wines, nuts, fruits, vegetables, blackcurrant, cocoa, cereals, and honey. Ali, et al. [40] reported that the molecular formula of anthocyanins is C_15_H_11_O^+^, whereas its molecular weight and melting point are 207.24 g/mol and 150 °C, respectively. Despite its high melting point, anthocyanins will degrade at a temperature of 80 °C [41], and it was also revealed that the degradation in anthocyanins was observed when treated at 80 °C. The anthocyanins structure links hydroxyl (−OH) and or methoxyl (−OCH_3_) groups and one or more sugars as shown in Figure 2 [42].

Table 2 shows that roselle contains 335.7 mg/g anthocyanins extracted using Soxhlet extraction with various different ratios of fresh roselle to water (1:10 g/mL), which were conducted using a water bath at a temperature of 60 °C for 60 min [43]. The anthocyanin concentration for roselle is higher than for strawberries and blueberries [10]. Although the anthocyanins concentration in roselle is lower than in cherries, this is due to the other isomerization of anthocyanins being dominant to increase the antioxidant activity [44].

Extraction is the separation of bioactive plant compounds from their inert components using selected solvents, which is the first step necessary to obtain the appropriate chemical components for further chemical analysis [24]. Pre-treatment, purification, and analysis are important steps in the extraction procedure. Traditional extraction methods are commonly categorized as a leaching process to separate extract from the solid (solid–liquid) [49,50]. Conventional methods include Soxhlet extraction and maceration extraction [51,52]. These techniques have been utilized for more than a century for the extraction, isolation, and purification of anthocyanins [29,30,42,53,54].

Anthocyanins are located in the inner cells of fruits and vegetables [55]. Acidic solvents are commonly used to denature the membranes of cell tissue and dissolve the pigments. Alappat, et al. [8] and Silva, et al. [30] reported that the acid has a tendency to stabilize anthocyanins, but it may also alter the natural form of pigment in the tissue by severing bonds with metals, pigments, or other variables. The recent research of solid–liquid extraction of anthocyanins from roselle calyces was performed by Wu, et al. [54]. They used 30% (*v*/*v*) ethanol in a 1:20 (*w*/*v*) ratio, followed by extraction at 75 °C for 20 min in a water bath, which resulted in about 361.99 mg/100 g of anthocyanins content. Meanwhile, the optimization model from the study by Miranda-Medina, et al. [56] showed that 96% ethanol, 65 °C, and a 1:50 calyx-to-solvent ratio gave the highest anthocyanins concentration of 150 mg/100 g.

## 3. Conventional Extraction of Anthocyanins

Conventional extraction of anthocyanins from roselle is nonselective and produces considerable quantities of by-products, such as sugars, organic acids, alcohols, organic acids, proteins, and amino acids [11,43,57]. Some of these contaminants, such as sugars, hasten the decomposition of anthocyanins during storage [58]. Efficiency in the extraction of anthocyanins can be maximized with the optimization parameter for a few degradation compounds. Additionally, the extraction technique should not be dangerous, too complex, time-consuming, or expensive. For the extraction of anthocyanins, methanol, ethanol, and acetone are more effective than using other solvents. However, solvents such as methanol/acetone may be hazardous to human health [59]. Therefore, the exploration of greener techniques for the extraction of anthocyanins is essential for current research [49].

Silva, et al. [30] have identified the benefits and drawbacks of anthocyanin extraction strategies using solid–liquid extraction, ScCO_2_, pressurized liquid extraction (PLE), ultrasound-assisted extraction (UAE), and microwave-assisted extraction (MAE). The solid–liquid extraction process involves a long extraction time, and within that time, exposure to acid, air, and light may cause anthocyanin degradation. In the UAE method, energy dissipation through heat may also cause anthocyanin degradation. Meanwhile, PLE and MAE require high temperatures during extraction which also contributes to degradation. The quality of natural extracts is significantly related to the extraction method, solvents used, vegetal matrix characteristics, and storage conditions. The selection of solvents must be carefully selected to maximize the yield and selectivity [29,60,61].

Even though ScCO_2_ extraction is a green extraction method, as it uses a nontoxic solvent, it is not suitable for anthocyanin recovery due to a difference in polarity between CO_2_ and anthocyanins. Currently, ethanol has been used as a co-solvent in order to enhance the polarity of CO_2_ [62,63]. Therefore, anthocyanins can be extracted using ScCO_2_ and ethanol as a co-solvent [11]. However, a greater concentration of co-solvent in the system would contribute to the solvent–solvent interactions in competition for the solvation of solutes, hence reducing the recovery [62].

## 4. ScCO_2_ as Green Extraction

Extraction is the process of separating constituents from natural substances using chemical or physical means. As a part of its efforts to conserve the environment, the global community has recently adopted green extraction [64,65]. ScCO_2_ as green extraction is based on methods that take less energy, permit the use of alternative solvents and renewable resources, and provide extracts that are both safe and high quality. It is regarded as the most effective alternative to conventional solvent extraction techniques for bioactive compounds. The four principles of eco-friendly natural product extraction are as follows [66,67]:

1. Renewable plant materials;
2. Use of alternative solvents and safe solvents;
3. Reduction in energy consumption;
4. Production of co-products as opposed to trash, including bio- and agro-refining industries.

To preserve natural materials, ScCO_2_ extraction requires either intensive culture or in vitro growth of plant cells or organisms. Natural colorants may be extracted from plants, animals, and fungi while preserving the rights of future generations. Using this method, it is possible to manufacture active components without solvent residue. CO_2_, which is widely utilized in the supercritical fluid extraction (SFE) technique, is an inflammable, odorless gas produced by the combustion of fossil fuels, the fermentation of alcohol, and human and animal respiration. In lieu of organic solvents such as hexane, the SFE approach employs compressed scCO_2_ at a pressure of up to 300 MPa and a temperature of 30 to 40 °C in the extraction process. Extraction is impacted by economic and environmental challenges, which need a significant reduction in energy use and waste generation.

Compared to conventional extraction procedures, the SFE approach expedites the extraction process. No further actions are necessary to save time or energy. A vast array of coproducts, by-products, and trash are generated during the extraction activities. According to the idea of biorefinery, plant resources are used in an integrated fashion. Plants contain several refined substances. Each component of a plant may be extracted and utilized to manufacture an assortment of goods. By creating functionalize co-products, the SFE process at low temperatures permits the identification of novel chemicals that improve the value of the extract. Reducing the number of steps in a manufacturing chain reduces costs and optimizes energy use. It indicates that the ideal approach is a one-step process. The advantages of supercritical fluid extraction include the use of a clean solvent and the production of an extract with minimum discrete processes. Furthermore, the extract is guaranteed to be harmless to people and the environment. Using this approach, the SFE method safeguards thermally unstable target molecules. In addition, the SFE extract has no residual solvent, making it very pure. Furthermore, the solvent of SFE can be recycled using a compressor, thus it will reduce the production cost [64].

## 5. ScCO_2_ as a Solvent for Anthocyanin Extraction

Carbon dioxide (CO_2_) has been used as a supercritical fluid for centuries. It is chosen based on its unique properties as discussed earlier. A comparison of its physical properties to some other fluids is shown in Table 3. Due to its low critical temperature, it can operate at moderate temperature, hence preventing substance degradation due to its heat induction [68]. CO_2_ is the preferred supercritical solvent for the extraction of bioactive compounds because it is colorless, odorless, non-flammable, inert, non-toxic, safe, recyclable, and can be utilized under mild operational conditions (critical temperature of 31.1 °C and critical pressure of 7.38 MPa). Furthermore, this method aims for high selectivity, high product purity, and low extraction time, while being non-hazardous to human health and the environment.

The main factors of a supercritical fluid are the critical pressure (Pc), critical temperature (Tc), the critical density and solubility of the fluid. The density of CO_2_ can be manipulated using different pressures and temperatures. Around the Pc, a significant change in density will occur in the enhancement of pressure. The solute solubility in a supercritical fluid is commonly influenced by the density of the solvent. Thus, a significant change in solubility is anticipated with an increase in pressure from the subcritical level (P < Pc) to the supercritical pressure level (P > Pc).

## 6. Parameter Effects on ScCO_2_ Extraction

Optimizing conditions to produce an adequate extraction of bioactive compounds while preventing undesired components is the initial stage of the ScCO_2_ procedure. Optimizing the operational parameters is a crucial stage in the development of the ScCO_2_ technology since numerous process factors might impact the extraction efficiency. Several SFE parameters, which may be adjusted [57], are required for the successful extraction of bioactive components from plant materials. Table 4 shows the optimal conditions to extract the rich anthocyanins from roselle.

Several factors have been proven to have significant effects on the efficiency of ScCO_2_ including the type of sample, fluid, pressure, flow rate, temperature, and extraction time. Reverchon, et al. [69] previously reported that all those parameters could affect the extraction rate. However, the main parameters that contribute to the extraction performance are pressure and temperature. In addition, the moisture content in the sample matrix and the sample and particle size as well as solvent flow rate will ultimately affect the extraction performance.

**Table 4:** The critical parameters in the ScCO_2_ extraction in the anthocyanin recovery.

Parameter	The Optimal Range	Ref.
Temperature	Temperature 40 to 70 °C	[11,70,71]
Pressure	Above 20 MPa	
Co-solvent	Ethanol (15%), combination ethanol and water	
Time	Less than 3 h	
Flowrate	1 to 10 mL/min, preferable slow flow rate to enhance the residence time of extraction	
Particle size	0.25 < dp < 1.80 mm	
Moisture content	4–14%	
Pre-treatment	Freeze-dried/sun dried	

### 6.1. Effect of Pressure at Constant Temperature in ScCO_2_ Extraction

Pressure is one of the important elements governing the ScCO_2_ process due to its influence on the solubility of compounds and diffusivity of the solvent [20,62,72]. Utilizing a back pressure regulator that maintains the desired pressure of ScCO_2_ may be used to conduct pressure control in the ScCO_2_ approach. At a fixed temperature, raising the pressure enhances the solubility of the bioactive compounds and the extraction yield by increasing the solvent power and extraction efficiency. In addition, increasing pressure enhances the density. As a result, the solubility improves, resulting in a greater recovery of anthocyanins [25]. If the pressure is raised above a certain point, the solvent diffusivity may decrease. Additionally, there may be less interaction with the pores of the raw material, which may lead to a reduction in solute dissolution [25]. On the other hand, a higher-pressure condition is not suggested for all targeted compounds as it might compress the raw material, hence decreasing the extraction yield [6]. The effect of pressure is also associated with the increase or decrease in solvent power due to the density of the solvent and by strengthening the intermolecular physical interaction [73].

### 6.2. Effect of Temperature at Constant Pressure in ScCO_2_ Extraction

The temperature has two distinct impacts at constant pressure. The enhancement of temperature decreases the density and solvating capacity of carbon dioxide. However, raising the temperature improves the solubility and extraction yield of the target compounds by increasing their vapor pressure. Consequently, this may result in the crossing of the isotherms, a process known as retrogradation in which higher-temperature conditions result in low yields and low temperatures result in a higher yield [11,20,25]. The inversion of yield isotherms is a result of these competing influences on the overall extraction yield.

By analyzing the crossover property, Cockrell, et al. [74] hypothesized that the density effect dominates at pressures below the crossover pressure, while the solute vapor pressure controls the extraction process at higher pressures. A greater temperature decreases the extraction recovery of nonvolatile substances. However, their solubility in ScCO_2_ is in competition with the volatility of their volatile components. The solubility of compounds alleviates as the temperature rises, although volatility increases. Although raising the temperature increases the extraction efficiency, many heat-sensitive compounds may break down or oxidize at higher extraction temperatures, losing their biological activity [52,75]. In order to prevent deterioration, the temperature condition for thermolabile compounds, especially for anthocyanins, must be adjusted between 35 and 60 °C [11]. The effects of temperature and pressure are related to each other. The increase in temperature at constant pressure will decrease solvent density and its solvation power [76]. The rise in temperature also will results in increasing solute vapor pressure, subsequently enhancing the solubility of a solute in the ScCO_2_.

### 6.3. Effect of Co-Solvent/Modifier in ScCO_2_ Extraction

A co-solvent is described as an organic solvent that may dissolve in ScCO_2_ at varying ratios and maintain a substantial degree of solvent power toward the targeted compounds [77]. Due to its intrinsic polarity, scCO_2_ is an ideal solvent for the extraction of nonpolar compounds, for example. However, pure CO_2_ is seldom used to extract hydrophilic compounds [78]. It is standard procedure to change the polarity of the ScCO_2_ by adding small quantities of organic co-solvents in order to boost the solvating power towards the target molecules. Strong polarity-dependent interactions, such as hydrogen-bonding and dipole–dipole interactions, between co-solvents and bio-components, such as hydrogen-bonding and dipole–dipole interactions, greatly improve extraction yields [79]. When finding the best co-solvent for a given extraction technique, the kind of material, targeted compounds, and preparatory tests should be considered [72]. Ethanol and methanol are the organic solvents most typically utilized [59]. According to the Food and Drug Administration (FDA) of the United States, ethanol is widely accepted as safe, making it the recommended co-solvent [70]. Due to its toxicity, methanol is not employed in the manufacturing of food or oil, despite the fact that it is used in ScCO_2_ processes on an analytical scale.

### 6.4. Effect of Particle Size in ScCO_2_ Extraction

Particle size is also a significant factor that contributes to the success of ScCO_2_. According to Reverchon, et al. [69], particle size affects to the mass transfer kinetics especially the diffusivity process and the contact of CO_2_ to the soluble compounds. Higher extraction efficiencies and the mass transfer surface can be influenced by the small physical morphology of the sample matrix, resulting from shorter internal diffusion path lengths over which the extracted solutes move to the fluid phase [80]. Generally, for natural products, the particle size varies from 0.25 to 1.80 mm; therefore, this issue must be evaluated case by case.

Another aspect that must be addressed is the particle’s form. As a result of the grinding process, the particles may be spherical, flaky, plate-like, or of various forms that will affect the diffusivity of the solvent in order to penetrate the raw material [81]. This trend is supported by the observation that decreasing the particle size increased the diffusivity coefficient of *Momordica charantia* extract in the range from 0.2 to 0.7 mm, thus the extraction yield increased [82]. On the other hand, the smallest particle size (0.2 mm) gives the lowest diffusivity coefficients (0.72 × 10^−13^ m/s^2^). This is because a smaller particle size will cause compaction and channeling inside the bed. However, fine particle size may result in bed caking and the formation of the preferential CO_2_ flow path, hence reducing the mass transfer. Therefore, it will reduce the anthocyanin recovery from roselle.

### 6.5. Effect of Flowrate in ScCO_2_ Extraction

CO_2_ flow rate is another important factor that needs to be considered while conducting the experiment. Machmudah, et al. [68] reported that enhancing the CO_2_ flow rate not only minimized the residence time but also enhanced the number of particles in contact with the anthocyanins, thus enhancing the intermolecular interaction between CO_2_ and the anthocyanins. Idham, et al. [25] also stated that the mass transfer of the extraction, which may be subdivided into a solubility-controlled zone and a diffusion-controlled phase, is highly influenced by the flow rate. A reduced solvent flow enhances the effectiveness, especially in the solubility-controlled area, and reduces the total solvent mass used to extract a given quantity of anthocyanins as interest compounds. As predicted, the anthocyanin production was directly related to the quantity of CO_2_ required for extraction in the solubility-controlled zone at lower pressures.

### 6.6. Effect of Extraction Time in ScCO_2_ Extraction

The extraction time relies on the types and conditions of raw material. It is also related to the other parameter such as pressure, temperature, size of particle and modifier. Generally, the extraction rate is increased as the time increased for short extraction time. However, towards the end of the extraction, the rate become slower due to transition to a diffusion controlled process from solubility controlled [83]. In addition, shorter time usage will contributes to the less consumption of CO_2_. Hossain, et al. [84] stated that increasing of extraction time is not significant to enhance the palm kernel cake oil recovery. Salajegheh, et al. [85] also stated that extraction efficiency of cocoa butter extract increases significantly up to 6 hours and then remain unchanged up to 8 h because the system reaches the equilibrium state.

## 7. Previous Studies of Anthocyanin Recovery from Roselle Using Scco_2_ Extraction

The presence of anthocyanin pigments in roselle is modest. Therefore, a particular extraction method is necessary to separate the dye-bearing components from their substrates as shown in Table 5 [74]. To obtain high yields and purity while keeping functional qualities such as color and bioactivity, the parameters of ScCO_2_ extraction have to be defined. Anthocyanins were extracted from roselle calyces using ScCO_2_ according to Idham, et al. [11]. Using a three-factor design, three processing parameters, namely pressure, temperature, and co-solvent ratio (ethanol–water), were examined. The approach was used to simulate the extraction of anthocyanins and their color properties. At maximum anthocyanins, the optimal conditions were 27 MPa, 58 °C, and an 8.86% co-solvent ratio. The production of anthocyanins was 1197 mg/100 g of dried roselle. Temperature is a significant factor to enhance the total yield of anthocyanins and color stability.

Idham, et al. [25] also discovered that the examination of the solubility yield and anthocyanin content of roselle extract at various temperatures of 50 to 70 °C, pressures of 8 to 12 MPa, and modifier ratios of 5 to 10%. This research demonstrated that a low temperature and high pressure at a constant modifier ratio enhanced the solubility of anthocyanins. Furthermore, the increasing temperature, pressure, and modifier ratio also increased the anthocyanin recovery.

Idham, et al. [41] also discovered that ScCO_2_ extraction was conducted at constant pressure and temperature, 10 MPa and 70 °C, respectively, using 75% (*v*/*v*) ethanol. The data indicates that 120 min was the optimal extraction duration for determining the flow rate. An increase in extraction time did not increase the anthocyanin recovery, but it would increase the production cost due to large CO_2_ consumption during the extraction process. These findings indicate that a low flow rate of 4 mL/min, a smaller particle size of 200 < dp < 355 µm, and a modifier percentage ratio of 10% might increase anthocyanin concentration. These process parameters might increase the selectivity of anthocyanin extraction.

Idham, et al. [86] also discovered that ScCO_2_ extraction of the red color of roselle was conducted using ethanol at pressures of 8 and 12 MPa, temperatures of 50 and 70 °C, and modifier flow rates between 5 and 10%. The total flow rate of CO_2_, modifier (6 mL/min), the ratio of the modifier (75% ethanol-25% water), the particle size utilized (dp < 350 µm), and the extraction time (70 min) were kept constant. The results indicated that the extraction yield was substantially impacted by the three primary influences examined in this research. The optimal working parameters for ScCO_2_ extraction of red color extract were 70 °C, 8.90 MPa, and 9.49%, with a red extract yield of 26.73%.

The optimal particle size (212 to 710 µm) was identified by Peng, et al. [87] to obtain the maximum total yield. Using the response surface methodology, the effects of two factors, pressure (20 to 30 MPa) and temperature (40 to 80 °C), on extraction yield were investigated. Based on the experimental results, the optimal particle size, pressure, and temperature were determined to be 300 µm, 27.5 MPa, and 50.8 °C, respectively. The greatest extraction yield estimated with the optimized parameters was 163.26 mg-extract/g-dried material. The most significant factor in enhancing the roselle extract is temperature due to the degradation of anthocyanins at high temperature.

The phenolic and flavonoid compounds were also recovered from roselle. Rizkiyah, et al. [70] discovered that pressure, temperature, and flow rate were used as variables in roselle extraction with ScCO_2_. The best conditions, as determined using the response surface methodology, were, 40 °C, 20 MPa, and 4.875 mL/min, producing 14.11% yield of red extract, 816.16 mg/L of TFC, and 935.48 mg/L of TPC, respectively. Pressure and temperature were significant factors that enhanced the roselle extract with ScCO_2_.

## 8. Comparison of ScCO_2_ Extraction and Traditional Extractions

Conventional techniques for extracting anthocyanins from plant material are nonselective and produce pigment mixtures including considerable quantities of by-products, such as sugar alcohols, sugars, amino acids, organic acids, amino acids, and proteins. Some of these contaminants hasten the decomposition of anthocyanins during storage [88]. Efficient separation techniques should optimize anthocyanin recovery with few degradations or changes to its natural form. Additionally, the extraction technique should not be too complicated, dangerous, time-consuming, or expensive. For the extraction of anthocyanins, using solvents such as ethanol, methanol, and acetone is more effective than using other solvents. However, solvents such as methanol/acetone may be hazardous to human health [52].

Maceration, Soxhlet extraction, and hydro distillation are frequently employed to extract bioactive compounds and natural pigments from a range of natural sources. The extraction temperature affects the effectiveness of the traditional extraction procedures. Although traditional procedures are straightforward, affordable, and simple to use, they need vast quantities of hazardous organic solvents, lengthy extraction durations, and additional solvent clean-up processes as shown in Table 6. Low purity and a lack of selectivity may lead to pigment degradation and unfavorable compound extraction as a result of the long process required by conventional procedures. Pigments have traditionally been extracted using organic solvents such as hexane, chloroform, isopropanol, acetone, and methanol. Due to the polar to nonpolar characteristics of the majority of pigments, mixtures of solvents such as n-hexane are used.

The high pressure required for the ScCO_2_ extraction process results in high capital and operations costs due to little or no toxic solvent. It is highly efficient, suitable for heat-sensitive pigments, and has a quick mass transfer (Table 6). ScCO_2_ is a nonpolar solvent with a predilection for nonpolar or low-polarity compounds, such as carotenoids [91]. Therefore, its applicability for the extraction of polar pigments, such as anthocyanins, is limited. In this situation, increasing its affinity needs the addition of a co-solvent to convert supercritical CO_2_ from a nonpolar to a polar solvent. Higher pressure increases the solvation power of CO_2_ (exhausted process) but reduces the selectivity of CO_2_ for compounds of interest, particularly anthocyanins.

## 9. Future Perspective

Numerous aspects of natural colorants need more investigation, and the present goal for scientists is to extract natural pigments from diverse sources, such as food sector leftovers, utilizing ecologically acceptable procedures to achieve low production cost and safety. Natural plant dyes may be in short supply due to limited resources. Therefore, roselle as a natural product has drawn the interest of scientists since it is seen as a possible source for novel natural high-yield dyes with extremely wide potential. In this respect, researchers are working to identify and validate the safety of red natural pigments; however, regulatory approval for these products is costly and time-consuming. Therefore, only a few naturally occurring colorants that are commercially accessible, such as anthocyanins, are FDA-approved for use in foods and drinks. As was previously stated, the restricted stability and poor solubility of natural colors in the application medium limit their employment in the food and pharmaceutical sectors. Alternative formulation procedures based on ScCO_2_ improve the solubility of anthocyanins. In addition, research was conducted on co-pigments and encapsulating methods to improve their hyperchromic effect, stability, solubility, and bioavailability. Co-pigments have been used to enhance the color and stability of a material. In the textile business, the anthocyanins derived from roselle are good for eczema sufferers. Nonetheless, the use of naturally colored textiles for wound healing has not yet been examined, and more study is required. Furthermore, ethyl acetate (EAc) can be applied as a substitute to extract anthocyanins from roselle in future research.

## 10. Conclusions

According to earlier research, ScCO_2_ extraction is a viable alternative to conventional extraction techniques that adds value by recovering high-quality pigments in an environmentally friendly way. This method has been utilized to generate unique natural red colorants, which has led to the creation of innovative concepts for the largest range of applications for these substances. Due to its pressure-tenable dissolving power, easy recovery, high selectivity, and nontoxicity, it is regarded as a green method for extracting natural antimicrobial colorants from different sources. ScCO_2_ parameters depends on anthocyanin recovery including temperature, pressure, flowrate, particle size, extraction time, and modifier ratio. The best parameters for the ScCO_2_ extraction of anthocyanins from roselle also influences the color’s stability. In many sectors, natural colorants extracted with ScCO_2_ are regarded as safe compounds, especially those meant for human consumption, such as novel functional food additives and textile and pharmaceutical colors.

## Figures and Tables

**Table 1:** Scientific reports on the toxicity of synthetic colorant adapted by Castro, et al. [33].

Colorant	Effect on Human Health	Ref.
Patent blue V	Allergic	[34]
Yellow twilight	Mutagenic	[35]
Tartrazine	Carcinogenic	[36]
Amaranth	Mutagenic, cytotoxicity, carcinogenic	[37]
Erythrosine	Mutagenic, cytotoxicity, genotoxicity	[38]
Red 40	Stomach, lung, kidney, and colon diseases	[39]

**Table 2:** Anthocyanins concentration from other plants.

Plants Material	Anthocyanins (mg/g)	References
Roselle	335.7	[43]
Strawberry	84.6	[10]
Blueberry	57	[9]
Fresh Plums	26–52	[45]
Cherry	50	[46]
Pomegranate	24	[47,48]

**Table 3:** Critical temperature and pressure of solvents.

No.	Solvents	Critical Temperature, Tc (°C)	Critical Pressure, Pc (atm)	Critical Density (g/mL)	Relative Polarity
1	CO_2_	31.3	72.9	0.448	0.01
2	C_2_H_6_0	241	63	0.235	0.654
3	H_2_O	374.15	218.3	0.315	1
4	C_6_H_14_	234.5	20.30	0.45	0
5	CH_4_O	−98.1	78.5	0.2	0.762

**Table 5:** Previous studies of anthocyanin recovery from roselle using ScCO_2_ extraction.

Parameter	Outcomes	Source
P = 27 MPa, T = 58 °C Co-solvent ratio = 8.86%	The production of anthocyanin was 1197 mg/100 g of dried roselle calyces.	[11]
T = 50 to 70 °C P = 8 to 12 MPa, Co-solvent ratio = 5 to 10%	A low temperature and rise in pressure at a constant modifier ratio would enhance the solubility of anthocyanin content	[25,41]
F_CO2_ = 4 mL/min Particle size = 200 < dp < 355 µm Co-solvent ratio = 10%	A low flow rate, smaller particle, and higher modifier percentage ratio of 10% might increase anthocyanin concentration	[86]
Particle size = 212 < dp < 355 µm T = 40 to 80 °C P = 20 to 30 MPa,	The optimal particle size, pressure, and temperature were determined to be 300 µm, 27.5 MPa, and 50.8 °C, respectively. The greatest extraction yield estimated with the optimized parameters was 163.26 mg/g. The most significant factor in enhancing roselle extract is temperature due to the degradation of anthocyanins at high temperature	[83]
P = 10 to 20 MPa, T = 40 to 60 °C F_CO2_ = 3 to 5 mL/min	The best conditions, as determined using response surface methodology, were 20 MPa, 4.875 mL/min and 40 °C producing 935.48 mg/L TPC, 14.11% red extract, and 816.16 mg/L TFC, respectively. Pressure and temperature were significant factors to enhance the roselle extract with ScCO_2_	[70]

**Table 6:** Comparison of the ScCO_2_ extraction with traditional methods.

Parameter	ScCO_2_ Extraction	Traditional Extractions	Ref.
Toxicity of Solvent	Safe	Harmful organic solvent	[11]
Volume of Solvent	Few	Large	[24]
Purity of extract	High	Low	[89]
Selectivity of solvent	High	Low	[52]
Extraction time	Fast	Slow	[75]
Solubility	High due to high pressure	Low (low pressure)	[1]
Cost	Expensive	Inexpensive	[90]

## Data Availability

Data available on request from the authors.
